# The Pivotal Immunomodulatory and Anti-Inflammatory Effect of Histone-Lysine N-Methyltransferase in the Glioma Microenvironment: Its Biomarker and Therapy Potentials

**DOI:** 10.1155/2021/4907167

**Published:** 2021-10-27

**Authors:** Seidu A. Richard, Kuugbee D. Eugene

**Affiliations:** ^1^Department of Medicine, Princefield University, P. O. Box MA 128, Ho, Ghana; ^2^Department of Molecular Medicine, School of Medicine and Dentistry, C.K. Tedam University of Technology and Applied Sciences, Navrongo, UER, Ghana

## Abstract

Enhancer of zeste homolog 2 (EZH2) is a histone-lysine N-methyltransferase that encrypts a member of the Polycomb group (PcG) family. EZH2 forms a repressive chromatin structure which eventually participates in regulating the development as well as lineage propagation of stem cells and glioma progression. Posttranslational modifications are distinct approaches for the adjusted modification of EZH2 in the development of cancer. The amino acid succession of EZH2 protein makes it appropriate for covalent modifications, like phosphorylation, acetylation, O-GlcNAcylation, methylation, ubiquitination, and sumoylation. The glioma microenvironment is a dynamic component that comprises, besides glioma cells and glioma stem cells, a complex network that comprises diverse cell types like endothelial cells, astrocytes, and microglia as well as stromal components, soluble factors, and the extracellular membrane. EZH2 is well recognized as an essential modulator of cell invasion as well as metastasis in glioma. EZH2 oversecretion was implicated in the malfunction of several fundamental signaling pathways like Wnt/*β*-catenin signaling, Ras and NF-*κ*B signaling, PI3K/AKT signaling, *β*-adrenergic receptor signaling, and bone morphogenetic protein as well as NOTCH signaling pathways. EZH2 was more secreted in glioblastoma multiforme than in low-grade gliomas as well as extremely secreted in U251 and U87 human glioma cells. Thus, the blockade of EZH2 expression in glioma could be of therapeutic value for patients with glioma. The suppression of EZH2 gene secretion was capable of reversing temozolomide resistance in patients with glioma. EZH2 is a promising therapeutic as well as prognostic biomarker for the treatment of glioma.

## 1. Introduction

Gliomas are primary brain malignant tumors which are often triggered by malignant modification of neural stem cells, progenitor cells, and differentiated glial cells such as astrocyte, oligodendrocyte, and ependymal cells [1–4]. These lesions are histologically grouped into Grades I-IV according to the World Health Organization (WHO) criteria [4, 5]. Most frequently, Grade I gliomas are detected in children and they mostly have good outcomes [1, 4]. However, Grade II gliomas are often associated with hypercellularity and have a 5-8-year average survival rate [4, 6]. Furthermore, Grade III comprises astrocytoma or anaplastic astrocytoma based on histological classification [4]. They are depicted with hypercellularity, nuclear atypia, and mitotic characters [4]. The anaplastic astrocytoma has a 3-year average survival rate [1, 7, 8]. Glioblastoma multiforme (GBM) comprises Grade IV gliomas [1, 4].

Enhancer of zeste homolog 2 (EZH2) is a histone-lysine N-methyltransferase that encrypts a member of the Polycomb group (PcG) family [9–11]. EZH2 is an enzyme that is encrypted by the EZH2 gene in humans. It is found on chromosome 7q35, and it contains 20 exons as well as 19 introns [9–11]. EZH2 is made up of multimeric protein complexes as well as associated with the preservation of the transcriptional suppressive state of genes over consecutive cell productions [9–11]. EZH2 is the catalytic subunit of the Polycomb repressive complex 2 (PRC2) which mediates the suppression of target genes that are associated with essential cellular processes via trimethylation of histone H3 on Lys 27 (H3K27me3) [12, 13].

EZH2 forms a repressive chromatin structure which eventually participates in regulating the development as well as lineage propagation of stem cells and glioma progression [12, 14]. Furthermore, EZH2 is involved in glioma initiation and progression as well as in the formation, maintenance, and plasticity of GSCs [15]. Thus, EZH2 may be a possible biomarker and therapeutic target in gliomas as well as in the development of novel treatment schemes that target both the genetic and epigenetic mechanisms of gliomagenesis. Further, the up- and downregulation of the EZH2 in *in vitro* as well as *in vivo* studies may be of a diagnostic as well as therapeutic biomarker in glioma treatment.

This review explores the fundamental immune and inflammatory players regulated by EZH2. The “Boolean logic” was used to search for articles on the subject matter. Most of the articles were indexed in PubMed and PMC with strict inclusion criteria being the immunomodulatory and anti-inflammatory effect of EZH2 in the glioma microenvironment which may be of biomarker and therapeutic importance. Search parameters were EZH2 and/or the posttranslational modifications, microenvironment, signaling pathways, biomarker, and therapy in gliomas.

## 2. Polycomb Group

PcG proteins are fundamental epigenetic modulators which constitute transcriptional repressors as well as crucial modulators of cell fate in cancer development [16, 17]. PcG proteins initiate their repressive actions via the formation of two distinctive protein multimeric complexes such as PRC1 and PRC2 in mammals [16, 18]. The PRC1 configuration is usually inconstant, and the mammalian core PRC1 is made up of B cell-specific Moloney murine leukemia virus integration site 1 (BMI1), ring finger protein (RING) 1 proteins such as RING1A and RING1B, chromobox (CBX), polyhomeotic (PH) proteins like PH1 and PH2, nervous system Polycomb 1 (NSPC1), or Polycomb group ring finger (Pcgf) 1 and Pcgf2 (MEL18) proteins [16]. The core subunits of mammalian PRC2 are often made up of EZH2 or EZH1, embryonic ectoderm development (EED), suppressor of zeste 12 (SUZ12), retinoblastoma protein-associated protein 46/48 (RbAp46/48), AE binding protein 2 (AEBP2), Polycomblikes (PCLs), and Jumonji and AT-rich interaction domain containing 2 (JARID2) [16, 19].

## 3. EZH2

EZH2 is the catalytically active domain of the PRC2 complex that partakes in transcriptional repression of precise genes via trimethylation of lysine 27 and, to a slighter extent, lysine 9 of histone H3 [20, 21]. EZH2 is a highly maintained histone methyltransferase (HMTase) which is capable of stimulating H3K27me3 as well as inhibiting transcription and secretion of target genes mediating several fundamental biological processes such as cell cycle modulation, cell fate assessment, senescence, cell proliferation, differentiation, apoptosis, and glioma progression [16, 17, 22].

Anomalous EZH2 secretion was extensively associated with a comprehensive array of aggressive as well as metastatic malignancies with poor outcomes because it was a core epigenetic modulator [16, 23, 24]. Studies have demonstrated that EZH2-mediated H3K27me3 acts as a docking location for PRC1 chromodomain-containing protein CBX as well as accelerates the preliminary recruitment of PRC1 that catalyzes H2AK119ub to conserve a repressed state of target genes [16, 25–28]. This therefore indicates a common as well as classic modulatory model that PRC1 functions downstream of PRC2 [16].

The EZH2-mediated methyltransferase complex in the cytoplasm was capable of stimulating actin polymerization, cellular adhesion, and migration resulting in glioma dissemination [29, 30]. Studies have established that 3-deazaneplanocin A (DZNep), S-adenosylhomocysteine- (SAM-) competitive inhibitors like GSK343, GSK126, and EPZ-6438, and the stabilized *α*-helix of EZH2 peptide (SAH-EZH2) are the three types of potent EZH2 inhibitors [31–33]. The SAH hydrolase inhibitor DZNep triggers the buildup of SAH resulting in a by-product blockade of the SAM-dependent methyltransferase action like EZH2 [31–33].

SAM is the general methyl donor for HMTase reaction; SAM-competitive inhibitors are the key routes for EZH2 inhibition due to their high selectivity for EZH2 [31, 32]. Yu et al. established that the introduction of GSK343 in glioma cells expressively reduced H3K27 methylation as well as coprecipitation with EZH2-H3 in a time-dependent manner and decreased the quantities of core units of PRC2 [31]. Furthermore, GSK343 treatment in normal glioma cells not only reduced the protein quantities of EZH2 but also downregulated the secretion of c-MYC [31].

## 4. Posttranslational Modifications

Posttranslational modifications (PTMs) are covalent processing actions that transform the structure and function of a protein via the proteolytic cleavage as well as addition of a modifying group, like acetyl, glycosyl, methyl, and phosphoryl, to one or more amino acids [16, 34, 35]. PTMs are often revisable or irreversible, and they participate in several critical biological processes by expressively affecting the structure as well as dynamics of proteins [16, 35]. PTMs often influence several protein behaviors as well as characteristics such as enzyme function and assembly, protein-protein interactions, protein lifespan, protein solubility, protein folding, protein localization, cell-cell as well as cell-matrix interactions, molecular trafficking, and receptor activation [16, 35].

PTMs are distinct approaches for the adjusted modification of EZH2 in the development of cancer [16]. E2 factors (E2Fs) are capable of binding to the promoter of EZH2 resulting in the transactivation of its secretion at the transcriptional level [16, 36]. The amino acid succession of EZH2 protein makes it appropriate for covalent modifications, like phosphorylation, acetylation, O-GlcNAcylation, methylation, ubiquitination, and sumoylation [37]. Thus, the most studied EZH2 PTMs include acetylation, phosphorylation, ubiquitination, sumoylation, and O-GlcNAcylation [16, 38–42].

**Figure 1:**
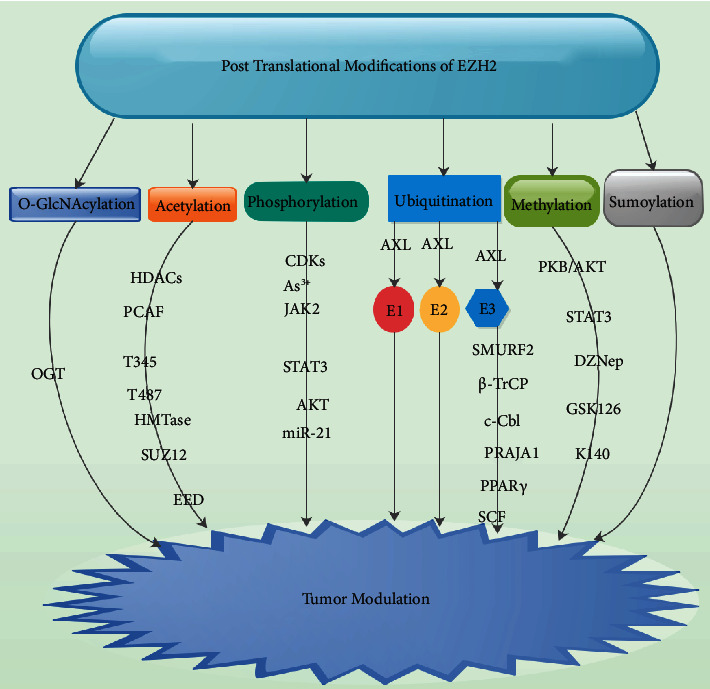
The posttranslational modifications of EZH2 and cascades.

Acetylation is a reversible and key type of PTM which involves the modulation of gene secretion primarily via the regulation of core histone tails by histone acetyltransferases (HATs) or histone deacetylases (HDACs) (Figure 1) [16, 43, 44]. Acetylation influences a series of cellular processes such as proliferation, apoptosis, differentiation, metabolism, and transcriptional modulation [45, 46]. Wan et al. demonstrated that EZH2 was acetylated by acetyltransferase P300/CBP-associated factor (PCAF) and was deacetylated by deacetylase SIRT1 (Figure 1) [39]. It was further established that PCAF was capable of interrelating with EZH2 resulting in the acetylation of EZH2 mainly at lysine 348 (K348) which triggers a reduction in EZH2 phosphorylation at T345 as well as T487 and augments EZH2 stability without either altering its interaction with other PRC2 complex members such as SUZ12 and EED or influencing its site and HMTase activity (Figure 1) [16].

O-GlcNAcylation refers to protein glycosylation with *β*-N-acetyl-D-glucosamine which is a reversible as well as a dynamic PTM activity universally observed in both the cytosol and the nucleus [47, 48]. It was established that EZH2 was capable of interrelating substantially with OGT as well as OGT-dependent O-GlcNAcylation of EZH2 at serine 75 (S75) which was necessary for the conservation of EZH2 protein stability and successive formation of H3K27me3 resulting in tumorigenesis (Figure 1) [42]. It was further demonstrated that O-GlcNAcylation of EZH2 at S75 inhibits phosphorylation at the same location obligatory for EZH2 degradation or shields EZH2 from other modifications at other locations that are advantageous for EZH2 degradation [16].

Phosphorylation normally transpires when protein kinases insert phosphate groups in an ATP-dependent approach to serine (Ser), threonine (Thr), tyrosine (Tyr), and histidine (His) residues of substrates, which triggers a conformational modification in the structure of several proteins resulting in their activation or deactivation and thus creating differences in the biological properties of their targets as well as binding affinities [49, 50]. Studies have demonstrated that phosphorylation of EZH2 at serine 21 (pS21 EZH2) was possible under different conditions. Signal transducer and activator of transcription (STAT) 3 S27 phosphorylation was capable of triggering arsenic- (As^3+^-) mediated growth stimulation via JNK pathways which resulted in AKT activation via upregulation of the negative AKT modulator miR-21 leading to pS21 EZH2 as well as oncogenesis (Figure 1) [16, 51, 52].

As^3+^-stimulated pS21 EZH2 was principally localized in the cytoplasm contrary to the notion that EZH2 was mainly a nuclear protein (Figure 1) [16, 51, 52]. Studies are needed on the role of As^3+^ initiation of AKT-dependent pS21 EZH2 via the stimulation of the JNK-STAT3-AKT signaling axis in glioma. Chen et al. demonstrated that EZH2 contains one perfectly matched (Thr350) and two imperfectly matched (Thr421 as well as Thr492) CDK phosphorylation motifs (K(R)S(T)PXK(R)) which are extremely evolutionally preserved from fruit flies to humans [16, 53]. Furthermore, mutation of Thr350 to alanine (T350A) led to about 60% decrease in CDK1-mediated EZH2 phosphorylation, while only about 30% or no decrease in phosphorylation was detected in T421A as well as T492A mutants which signify that Thr350 was a major CDK-mediated phosphorylation site [53].

It was established that JAK2-stimulated phosphorylation at EZH2 Y641 (pY461 EZH2) triggered EZH2-*β*-TrCP intercommunication resulting in *β*-TrCP-mediated EZH2 ubiquitination as well as proteasomal degradation which triggered downregulation of EZH2 protein stability as well as H3K27me3 hypoactivity, signifying phosphorylation-dependent EZH2 ubiquitination [16, 54, 55]. In ubiquitination, Ub covalently attaches to the modified proteins and modulates their stability and functions as well as localizes their involvement in several cell functions as well as diseases, expressly in cancer development [54, 55].

Ubiquitination transpires via the stimulation of a cascade of enzymatic reactions dependent on three obligatory enzymes like ubiquitin-activating enzyme (E1), ubiquitin-conjugating enzyme (E2), and ubiquitin ligase (E3) (Figure 1) [16, 54, 55]. Microarray studies of U87MG glioma cells after EZH2 silencing revealed a robust transcriptional reduction of the AXL receptor kinase [56]. Histone modification was associated with the positive modulation of AXL by EZH2 [56, 57]. The knockdown of AXL imitated the anti-invasive properties of EZH2 silencing, and AXL secretion was detected in human gliomas with elevated EZH2 secretion [56, 57].

Studies have demonstrated that SMAD ubiquitination regulatory factor-2 (SMURF2), *β*-TrCP (FBXW1), Casitas B-lineage lymphoma (c-Cbl) protein, and PRAJA1 function as dynamic EZH2 ubiquitin E3 ligases (Figure 1) [40, 58–60]. Ub E3 ligase PRAJA1 triggered ubiquitination-proteasome pathway-mediated EZH2 protein degradation [59]. Also, SMURF2 interacts with EZH2 resulting in the stimulation, ubiquitination, and proteasome-mediated degradation of EZH2 at lysine 421 leading to upregulation of its target gene PPAR*γ* (Figure 1) [40]. A recent study established that EZH2 acts as a substrate for Skp/cullin/F-box protein (SCF) and ubiquitin E3 ligase *β*-TrCP (Figure 1). Furthermore, EZH2 was expressly interrelated with *β*-TrCP resulting in *β*-TrCP-mediated EZH2 ubiquitination [58].

Sumoylation is an extremely preserved enzymatic cascade in which a tiny ubiquitin-like modifier (SUMO) protein is enzymatically conjugated to the *ε*-amino group of certain lysine residues [61]. Sumoylation was primarily authenticated to be linked to the modulation of EZH2 activity (Figure 1) [41]. It was established that EZH2 had several SUMO-modified locations or diverse configurations of sumoylation on the same location because EZH2 displayed several bands of modifications in both western blot analysis and *in vitro* sumoylation assay [16].

DNA methylation is an enzyme-mediated chemical modification of DNA by the insertion of a methyl group from S-adenosyl-L-methionine substrates to the 5-position of cytosine (5-methylcytosine (5mC)) [56, 62]. It was established that DNA methylation can directly modulate gene secretion by repressing the binding of fundamental transcription factors as well as by indirectly recruiting methyl-CpG-binding domain (MBD) proteins to the promoter gene (Figure 1) [56]. Studies have demonstrated that EZH2 was capable of modulating oncogenic gene secretion by mediating the DNA methylation level [9, 63]. Furthermore, EZH2 was capable of methyltransferase activity and the oversecretion of EZH2 was capable of modulating transcription of downstream genes via DNA methylation [20].

Protein kinase B (PKB)/AKT-induced pS21 EZH2 was capable of accelerating EZH2-STAT3 intercommunication (Figure 1), stimulated EZH2-mediated STAT3 methylation, and augmented STAT3 activity in glioblastoma multiforme (GBM) stem-like cells (GSCs) [64]. This indicates that the AKT-pS21 EZH2-STAT3 signaling axis is a prospective modulator of GSC tumor malignancy and an auspicious therapeutic target for GBM [64]. Furthermore, EZH2 binds to and methylates STAT3, leading to augmented STAT3 activity via upregulation of tyrosine phosphorylation of STAT3 [64].

Kim et al. demonstrated that the EZH2 blockade significantly reduced universal levels of H3K27 trimethylation and p-STAT3 in GSCs [64]. Also, p-STAT3 in GSCs was precipitously reduced when either DZNep or GSK126 was introduced (Figure 1) [64]. They detected oversecretion of EZH2 S21 stimulated STAT3 methylation as well as augmented STAT3 activity [64]. They concluded that EZH2 S21 phosphorylation was a molecular switch that accelerates STAT3 methylation [64]. Yang et al. demonstrated that K140 methylation of STAT3 destabilizes STAT3 tyrosine phosphorylation resulting in a negative influence on STAT3-dependent transcription [65].

It was further established that K140 methylation functions as a negative influence in the STAT3 signaling cascade which is directly opposite to K180 STAT3 methylation by EZH2 [64]. Ott et al. indicate that EZH2 stimulates transcription of AXL mRNA in a methylation-independent manner [57]. It was established that H3K27M blocked the enzymatic action of the PRC2 via communication with the EZH2 subunit [56]. Transgenes comprising lysine-to-methionine substitutions at other known methylated lysines like H3K9 and H3K36 are adequate to trigger a specific decrease in methylation via the blockade of SET-domain enzymes [56].

## 5. EZH2 and Glioma Microenvironment

The glioma microenvironment is a dynamic component that comprises, besides glioma cells and GSCs, a complex network that comprises diverse cell types like endothelial cells, astrocytes, and microglia as well as stromal components, soluble factors, and the extracellular membrane (ECM) [2, 66–68]. Furthermore, glycolytic metabolism was almost 3 times higher in normal brain tissue compared to GBM and was modulated by oncogenes like phosphoinositide 3-kinase (PI3K), AKT, and hypoxia-inducible factor 1 (HIF1) [69, 70]. Studies have shown that BGB324 and BMS-777607 are targets for AXL that suppressed multiple malignant activities like growth, migration, and invasion in GBM [71–73].

Ott et al. demonstrated that the blockade of EZH2 decreased glioma cell proliferation as well as invasiveness [57]. They indicated that EZH2 triggers glioma invasiveness via transcriptional regulation of AXL (Table 1) [53]. Yen et al. revealed that n-butylidenephthalide (BP) targeting AXL (Table 1) decreased brain tumor migration and invasion as well as prolonged animal survival in orthotic GBM animal models [71]. They further disclosed that BP was capable of downregulating EZH2 secretion and inhibiting the secretion of AXL in a dose-dependent manner of GSCs [71]. Jin et al. demonstrated that EZH2 influenced hypoxia, acidic stress, and nutrient restriction which promoted GSC maintenance (Table 1) [74]. Moreover, the RNF144A-BMI1 regulatory mechanism was capable of empowering GSCs to reside in stressful microenvironments [74].

Studies have shown that BMI1 was capable of modulating tumor induction and growth in a genetically engineered murine model of GBM as well as human stem-like glioma lines [74–77]. Also, BMI1 binds and modulates the promoters of several genes, including TGF-*β*, which was intensely associated with the mesenchymal phenotype [74, 78]. GBM cells are capable of experiencing molecular subtype transitions under the influence of a diverse tumor milieu resulting in diverse consequences between the *in vitro* and *in vivo* experiments by BMI1 and EZH2 inhibitor administration (Table 1) [74]. Studies have shown that STAT3 signaling was capable of modulating mesenchymal transformation of gliomas [64]. Furthermore, STAT3 downstream genes were extremely secreted in the mesenchymal GBM subtype [64, 79].

Studies further revealed that INK4B-ARF-INK4A, which encodes three distinct proteins, *p15^INK4b^*, *p14^ARF^*, and *p16^INK4a^*, p57, bone morphogenetic protein receptor 1B (BMPR1B), MyoD, and RUNX3 are all negatively modulated by EZH2 (Table 1) [69, 80, 81]. Liu et al. also established that the E-cadherin gene (CHD1) (Table 1) is associated with epithelial-mesenchymal transition (EMT), invasion, and migration in an essential inhibitory target of EZH2 [82]. Furthermore, molecules like BIM, TNF-related apoptosis-inducing ligand (TRAIL), and FBO32 which are associated with apoptosis are suppressed by EZH2 (Table 1) [83, 84]. Lu et al. demonstrated that Vasohibin1, a molecule meticulously linked to tumor angiogenesis, was also suppressed by EZH2 [85]. Zhou et al. demonstrated that EZH2 and MICU1 were obligatory in conserving mitochondrial membrane potential stability (Table 1). Also, they were capable of modulating tumor growth via regulation of a mitochondrial-dependent cell-death pathway [86].

Pang et al. discovered that oxygen consumption rates were decreased in knockdown EZH2 GBM cells (Table 1), which indicates a deficiency in the TCA cycle [69]. Furthermore, oversecretion of EZH2 exerted a negligible influence on mitochondrial oxidative capacity [69]. However, the oversecretion of EZH2 triggered glycolytic metabolism which resulted in a significant increase in cellular deoxyglucose uptake as well as the activities of key enzymes associated with glycolysis and lactate production (Table 1) [69]. Thus, EZH2 was capable of modulating the Warburg effect in GBM [69].

Pang et al. further observed that exogenous oversecretion of EZH2 augmented HIF1*α* secretion under normoxia (Table 1) [69]. HIF1*α* modulation typically depends on oxygen-dependent protein stability [69]. It was established that HIF1*α* was hydroxylated by a family of oxygen-dependent prolyl hydroxylases (PHD1-3) resulting in the binding of pVHL to HIF1*α* for ubiquitination as well as proteasomal degradation under normoxic conditions (Table 1) [69]. Pang et al. also detected that the secretion of tumor suppressor protein EAF2 was repressed by EZH2 because the depletion of EZH2 correlated with the stimulation of EAF2 (Table 1) [69].

Wang et al. observed that EZH2 was crucial for glioma cell aerobic glycolysis [87]. They detected that the blockade of EZH2 activity by siRNA as well as DZNep reduced the magnitude of glycolysis under basal conditions (Table 1), the glycolytic capacity, and the glycolytic reserve [87]. Yu et al. established that GSK343, a blocker of EZH2 (Table 1), inhibits the proliferation, invasion, and cancer stem-like phenotypes and reverses mesenchymal transition of glioma cells *in vitro* as well as *in vivo* [31]. CDKN2A is a tumor suppressor gene that encodes for p16 protein and functions as a cellular senescence as well as a negative modulator of cell cycle progression. Several studies have detected CDKN2A deletion/loss of p16 protein secretion in high-grade gliomas [12, 88–90].

Purkait et al. detected that about 80% of samples with p16 loss with deficiency of CDKN2A homozygous deletion exhibited robust EZH2 secretion [12]. They suggest that EZH2 mediated downregulation of p16 secretion in the samples [12]. The mechanism of the EZH2-mediated blockade of p16 secretion was a result of the repressive chromatin mark H3K27me3 triggering the recruitment of the PRC1 complex. Studies have demonstrated that p16 acts as a negative modulator of cell cycle progression from the G1 phase to the S phase [91, 92]. Thus, high-grade glioma cells may elude the p16 cell cycle checkpoint either via the homozygous deletion of CDKN2A or via the EZH2-mediated transcriptional knockout of p16 protein secretion which means that loss of p16 protein secretion augments proliferative activity (Table 1) [12].

Wang et al. demonstrated that EZH2 staining was expressively dissimilar in epithelioid cells as well as low-grade sections of five biphasic epithelioid glioblastoma (EGBM) samples [93]. They further observed robust EZH2 secretion in epithelioid cells with a high Ki67 index but not in low-grade lesions which suggested that EZH2-positive cells are associated with intratumoral heterogeneity as well as the malignant progression of the tumor [93]. They also observed a coexistence of robust EZH2 secretion, BRAF V600E, and CDKN2A/B deletions in EGBM samples, but no negative correlations between robust EZH2 secretion and CDKN2A/B deletions were detected (Table 1) [93].

Yin et al. established that the EZH2 blockade in GBM stimulation promoted the elevation of M1 markers like iNOS and TNF-*α* as well as the decrease in a pool of M2 markers in murine microglia and human PBMC-derived macrophages (Table 1) [94]. They further observed that the EZH2 blockade in GBM cells augmented the phagocytic capabilities of cocultured microglia via the stimulation of iNOS [94]. Mechanic studies revealed that the knockdown of EZH2 blocked the secretion of anti-inflammatory factors while promoting the secretion of proinflammatory factors in GBM cells [94]. The EZH2 blockade in GBM facilitated the polarization shift of microglia as well as PMMC-derived macrophages resulting in an upsurge of M1 markers and a decrease of M2 markers [94].

Ahani et al. demonstrated that human cytomegalovirus (HCMV) gene products are capable of facilitating the PI3K/AKT pathway which was associated with apoptosis, angiogenesis, invasion, and immune evasion resulting in tumor growth [95]. They further established that HCMV was capable of facilitating the progression of GBM via upregulation in the secretion of the EZH2 gene because they observed oversecretion of EZH2 in HCMV-positive GBM models compared to HCMV-negative GBM models (Table 1) [95]. Bioinformatics analysis revealed that miR-133b was capable of influencing the EZH2 gene in glioma. EZH2 was aberrantly secreted in glioma as well as contributed to the invasive and metastatic capabilities of GBM [71].

## 6. EZH2 Signaling Pathways in Glioma

EZH2 oversecretion was implicated in the malfunction of several fundamental signaling pathways like the wingless-related integration site (Wnt)/*β*-catenin signaling, rat sarcoma (Ras) and NF-*κ*B signaling pathways, PI3K/AKT pathway, *β*-adrenergic receptor signaling, and bone morphogenetic protein (BMP) as well as NOTCH signaling pathways in cancers [16, 96–99]. The Wnt/*β*-catenin signaling pathway partakes in the development of the central nervous system and is linked to oncogenesis in many cancers [100, 101]. It was established that downstream of EZH2 had inhibitory effects on glioma growth via the blockade of the *β*-catenin signaling pathway [87, 100]. Also, the Wnt/*β*-catenin signaling pathway was associated with GBM progression [100, 102].

**Figure 2:**
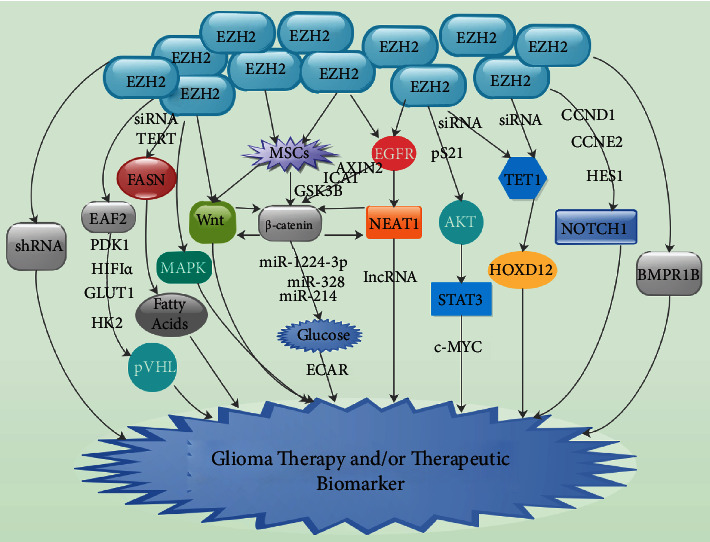
Signaling pathways via which EZH2 modulates the glioma microenvironment.

Domenis et al. demonstrated that exosomes derived from mesenchymal stem cells (MSCs) were capable of facilitating glioma development [103]. Xu et al. demonstrated that MSC-derived exosomes oversecreting miR-133b were capable of blocking the progression of glioma via the EZH2-Wnt/*β*-catenin signaling pathway [100]. They indicated that MSC-derived exosomal miR-133b was capable of blocking glioma cell proliferation, invasion, and migration as well as tumor growth via the suppression of EZH2 through the blockade of the Wnt/*β*-catenin signaling pathway in *in vitro* as well as *in vivo* experiments (Figure 2) [100]. The EZH2-miR-328/*β*-catenin signaling cascade could act as an innovative therapeutic biomarker for glioma. Also, inhibition of EZH2 was linked to the suppression of glioma growth via the inhibition of the *β*-catenin signaling pathway (Figure 2) [87, 100].

Chen et al. demonstrated that the EGFR/NEAT1/EZH2/*β*-catenin axis in GBM bestowed an oncogenic activity in GBM that is of novel therapeutic potential (Figure 2) [104]. Wang et al. detected that miRNAs like miR-1224-3p, miR-328, and miR-214 are repressed by EZH2 which was also modulated by *β*-catenin secretion via its 3′UTR in gliomas (Figure 2) [87]. They indicated that miR-328 served as a tumor inhibitor by abolishing EZH2 activities on glucose metabolism in glioma cells [87]. They further identified an EZH2/miRNA/*β*-catenin feedforward loop associated with the oversecretion of EZH2, *β*-catenin, and miRNA repression in glioma glucose metabolism (Figure 2) [87]. Juan et al. demonstrated that miR-214 negatively modulated EZH2 secretion by targeting the EZH2 3′UTR [105]. Thus, EZH2 and miR-214 form a modulatory loop controlling PcG-dependent gene secretion [87].

AKT-stimulated pS21 EZH2 was capable of accelerating EZH2-STAT3 intercommunication and augmenting EZH2-mediated methylation as well as activities of STAT3 resulting in the facilitation of GSC self-renewal as well as GBM tumor progression (Figure 2) [38]. It was demonstrated that EZH2 was capable of interacting with DNA methyltransferases (DNMTs) resulting in an influence on DNMT activity [56]. Furthermore, the binding of DNMTs to respective EZH2-repressed genes depended on the existence of EZH2 [56]. Cartron et al. demonstrated that EZH2 downregulation influenced the ten-eleven translocation 1 (TET1)/EZH2 intercommunications in U251 cells [106]. They further demonstrated that downregulation of EZH2 reduced TET1 recruitment on the HOXD12 genes [106]. They indicated that reduction of TET1/EZH2 recruitment on the HOXD12 gene in cells treated with siRNA-EZH2 generated an upsurge in methylation of the HOX12D gene (Figure 2) [106]. They concluded that EZH2 functions as an anchor for TET1 recruitment on the HOXD12 genes [106].

Chen et al. exhibited that administration of melatonin expressively influenced sphere morphology, EZH2-STAT3 intercommunications, and STAT3 activity in AKT1-oversecreted GSCs (Figure 2) [107]. They indicated that melatonin was capable of influencing the AKT-EZH2-STAT3 signaling axis resulting in robust impairment of GSC self-renewal as well as cancer-initiating capacity. Also, administration of melatonin blocked EZH2 S21 phosphorylation as well as EZH2-STAT3 intercommunication [107]. However, AKT1 oversecretion annulled this effect which means that AKT was a fundamental downstream effector of melatonin in GSCs.

A study established that PI3K/AKT signaling was augmented in about 90% of GBM samples [108]. Chen et al. observed that AKT oversecretion distinctly augmented EZH2 S21 phosphorylation concentrations, EZH2-STAT3 intercommunications, and STAT3 activity but downregulated H3K27me3 concentrations [107]. Thus, AKT-STAT3-EZH2 signaling and EZH2 phosphorylation participated in cancer stem cell (CSC) growth as well as carcinogenesis, and administration of melatonin blocked EZH2 S21 phosphorylation as well as EZH2-STAT3 intercommunications and modulated histone modifications resulting in the blockade of tumor initiation as well as propagation [107]. Yu et al. discovered that EZH2 participated in glioma tumor progression via EZH2-STAT3-c-MYC dependent pathways (Figure 2) [31].

Kim et al. established that the blockade of AKT signaling reduced STAT3 activity via EZH2 phosphorylation, indicating that PI3K/AKT signaling was an upstream modulator of the EZH2-STAT3 intercommunication in GSCs (Figure 2) [64]. They revealed that EZH2 S21 phosphorylation was obligatory for the EZH2-STAT3 intercommunication as well as augmentation of STAT3 activity, and the AKT blockade *in vivo* essentially stopped pS21 EZH2 (Figure 2) [64]. Thus, pS21 EZH2 secretion is a potential therapeutic biomarker via the PI3K/AKT axis [64]. Studies have shown that stimulation of the PI3K/AKT pathway in glioma was linked to an unfavorable clinical prognosis when various PI3K/AKT inhibitors were subjected to clinical trials [64, 108].

Zheng et al. established that melatonin extremely decreased NOTCH1 and other NOTCH1 signaling pathway components like CCND1, CCNE2, and HES1, which are modulated by NOTCH1 in GSCs (Figure 2) [109]. They observed that the active NOTCH1 protein portion and NOTCH intracellular domain 1 (NICD1) were deregulated, signifying that NOTCH1 was capable of mediating the activities of EZH2 upon administration of melatonin [109]. They further revealed that EZH2 modulated NOTCH1 secretion by directly interrelating with the NOTCH1 promoter [109]. They emphasized that a substantial correlation in the secretion of EZH2 and NICD1 was detected in tumor samples from GBM patients, signifying the existence of the EZH2-NOTCH1 signaling pathway in malignant gliomas [109].

Natsume et al. showed that biological interconversion between GSCs and differentiated non-GSCs correlated with the gain or loss of EZH2/PRC2-mediated H3K27me3 on pluripotent or development-related genes like NANOG, Wnt1, and BMP5 [110]. Pang et al. detected a substantial inverse correlation in the secretion of EZH2 and EAF2 which signifies that EAF2 influenced EZH2 activities [69]. Furthermore, EAF2 binds to and stabilizes pVHL resulting in a disruption of the HIF1*α*-mediated hypoxia signaling pathway (Figure 2) [111]. H3K27ac coinhibition augmented the efficacy of EZH2 inhibitors and also triggered the MAPK pathway in some cancers, signifying that the blockade of EZH2 resulted in a feedback stimulation of certain signaling pathways in a context-dependent manner (Figure 2) [112, 113].

NF-*κ*B is a major modulator of fundamental cell processes like inflammation, proliferation, and apoptosis [112, 114]. Several stimuli, comprising inflammatory cytokines like tumor necrosis factor-*α* (TNF-*α*) and interleukin-1*β*, which induce the classical pathway, are capable of triggering the NF-*κ*B signaling [112, 115]. It was established that SAH was able to trigger the stimulation of the NF-*κ*B pathway resulting in endothelial dysfunction as well as stimulation by partially blocking the enzymatic activity of EZH2 [112, 116]. Jiang et al. implicated SOX9 as a fundamental downstream target of EZH2 in rat cells [117]. Also, the blockade of EZH2 reduced the concentration of H3K27me3 at the SOX9 promoter region as well as augmented SOX9 secretion in rat endplate chondrocytes (EPCs) [112, 117].

Min et al. demonstrated that EZH2 triggered Ras and NF-*κ*B by epigenetically inhibiting DAB2IP, which stimulates the molecular mechanism through which an epigenetic modulator triggers these two major signaling pathways [26]. Studies on EZH2-Ras-NF-*κ*B signaling pathways in glioma are warranted. ADRB2 is a G protein-coupled receptor (GPCR) of the *β*-adrenergic signaling pathway. Yu et al. demonstrated that ADRB2 is a target for EZH2-mediated transcriptional repression [96]. Nevertheless, no studies on EZH2-ADRB2 signaling in glioma exist [96]. Thus, studies on EZH2-ADRB2 signaling in glioma are warranted. The hallmark of cAMP/*β*-adrenergic signaling is its capability of blocking cell proliferation in certain types of cells while activating cell growth in others [118]. Studies on EZH2-*β*-adrenergic receptor signaling in glioma are needed.

## 7. EZH2 as a Biomarker in Glioma

EZH2 is well recognized as an essential modulator of cell invasion as well as metastasis in glioma [71, 100]. It was established that EZH2 was more secreted in GBM than in low-grade gliomas as well as extremely secreted in U87 human glioma cells [42]. Orzan et al. demonstrated that EZH2 was upregulated in malignant gliomas [119]. They established that EZH2 secretion was 26.62 ± 19.90-fold elevated in 57 GBM specimens compared to normal brains [119]. They also evaluated EZH2 levels in nine low-grade gliomas and detected that the secretion of EZH2 was 4.26 ± 2.90-fold elevated compared to normal brains, which was significantly lower than that in GBM [119]. This signified that EZH2 secretion was linked to glioma malignancy [119].

Ott et al. observed a robust EZH2 secretion in GBMs while the secretion was low in Grade III astrocytoma as well as absent in Grade II astrocytoma [57]. They indicated that the knockdown of EZH2 suppressed glioma cell proliferation as well as invasiveness, and it also suppressed AXL receptor kinase secretion [57]. Wu et al. also detected elevated secretion of EZH2 in gliomas and suggested that EZH2 participated in the modulation of glioma development [120]. It was established that BMI1 and EZH2 secretion in glioma tissues were expressively elevated compared to those in nonneoplastic brain tissues [56]. Furthermore, upregulations of BMI1 and EZH2 proteins were both expressively associated with advanced WHO grades as well as low Karnofsky [56].

Wu et al. observed that the overall survival of patients with elevated BMI1 protein secretion or elevated EZH2 protein secretion was apparently lower than those with low secretions [120]. It was established that EZH2 was capable of stimulating aerobic glycolysis in tumors [121, 122]. Also, EZH2 was capable of switching mitochondrial respiration to glycolysis *in vitro* by augmenting the level of H3K27me3 at EAF2 promoter areas in GBM cells [121, 122]. This inhibited the transcription of EAF2 as well as triggered the HIF1*α* signaling pathway resulting in the transcription of downstream genes like HK2, glucose transporter 1 (GLUT1), and PDK1 which are associated with metabolism (Figure 2) [121, 122]. Thus, EZH2 was capable of accelerating tumorigenesis as well as the malignant progression of tumor cells via the stimulation of the Warburg effect [122].

Specifically, the blockade of EZH2 activity inhibited aerobic glycolysis in glioma cells [121, 123]. It was discovered that the glycolytic capability and reserve were both reduced when the concentrations of EZH2 are reduced in U87 as well as U251 glioma cells [121, 123]. EZH2 was capable of binding to the miR-328 promoter and downregulating miR-328 via a recognized H3K27me3 modification fashion [123]. Furthermore, miR-328 was also capable of blocking the secretion of *β*-catenin [121, 123]. Also, the EZH2/miRNA/*β*-catenin pathway triggered an upsurge in the extracellular acidification rate (ECAR) resulting in an augmentation in the glycolytic capability (Figure 2) [121, 123]. It was revealed that telomerase reverse transcriptase (TERT) and EZH2 jointly stimulated PCG-1*α* resulting in the secretion of fatty acid synthase (FASN) in glioma having (TERT) promoter mutations [121, 124].

Higher EZH2 concentrations in TERT mutants participated in gliomagenesis via epigenetic reprogramming of H3K27me3 modification marks because EZH2 silencing influenced not only TERT secretion but also lipid metabolism [121, 124]. Also, the pharmacological blockade of human TERT repressed the secretion of EZH2 as well as FASN and reduced the buildup of fatty acids [121, 124]. Nevertheless, reduced secretory levels of TERT as well as FASN and decreased levels of intracellular fatty acids were detected upon siRNA-mediated EZH2 silencing (Figure 2) [121, 124]. Thus, EZH2 endorses the synthesis of fatty acid as well as lipid buildup through the TERT-EZH2 pathway (Figure 2) [121, 124].

Fan et al. demonstrated that in experiments involving EZH2 siRNA and controls, the percentage of cells in the G1 phase exhibited a steady rising trend, while the percentage of cells in the S, G2, and M phases reduced concordantly, implying G1 arrest [9]. It was established that EZH2 siRNA was capable of blocking the progression of the cell cycle via the inhibition of transition from the G1 phase to the S and G2 phases [9, 56]. Furthermore, the silencing of EZH2 secretion by using RNA interference in U87 human glioma cells triggered apoptosis and cell cycle arrest in the G0/G1phase [9, 56]. Also, the knockdown of EZH2 modified the mitochondrial membrane potential as well as endorsed the expression of cytochrome c from the mitochondria [9, 56].

Zhang et al. established that decreased secretion of EZH2 modified Bax as well as Bcl-2 protein levels and triggered the stimulation of caspase 9 and caspase 3 [125]. Smits et al. demonstrated that the blockade of EZH2 *in vivo* by systemic DZNep treatment in a U87-Fluc-mCherry GBM xenograft mouse imaging model led to inhibition of tumor growth [126]. Wang et al. demonstrated that lncRNA transcribed from the 5-prime end of the HOXA transcript HOXA11-AS participated in the malignant progression of GBM [127]. Chen et al. established that nuclear enriched abundant transcript 1 (NEAT1) was a preserved lncRNA in diverse species and EZH2 was a hypothetical NEAT1-binding protein (Figure 2) [104].

Chen et al. specified that the GBM-linked lncRNA NEAT1 was an oncogenic factor that was modulated via the EGFR pathway and triggered tumorigenesis by acting as a scaffold as well as recruiting the chromosome modification enzyme EZH2 to knock down target-specific genes like AXIN2, ICAT, and GSK3B which facilitated *β*-catenin nuclear transport (Figure 2) [104]. Zheng et al. exhibited that EZH2 secretion correlated with GSC proliferation, self-renewal, and GSC marker secretion, signifying that EZH2 regulated the “stemness” of the GSCs [109]. They observed that melatonin reduced GSC viability or self-renewal to an analogous level in both the control and EZH2-oversecreted cells [109].

Purkait et al. demonstrated that EZH2 was not secreted by the normal brain, reactive glial tissue, and circumstantial nonneoplastic glia [12]. EZH2 was variably secreted in the nuclei of tumor cells in Grades II to IV gliomas [12]. They indicated that the secretion of EZH2 was slightly irregular, with a low labeling index (LI) in Grade II gliomas, while its secretion became more regular as well as widespread with high LI in higher-grade gliomas [12]. Their finding suggests that aberrant secretion of EZH2 was associated with malignant progression [12]. Moreover, EZH2 protein secretion correlated with mRNA secretion in their study [12]. Furthermore, they indicated that EZH2 immunohistochemistry was capable of differentiating nonneoplastic reactive glial proliferation from gliomas, thus showing its diagnostic application in routine neuropathology practice [12]. Also, high LI of EZH2 was a potential indicator supporting the diagnosis of higher-grade gliomas like Grades III and IV gliomas [12].

## 8. EZH2 and Glioma Therapy

EZH2 is both a promising therapeutic target and a prognostic factor in brain tumors [56, 128]. It was established that *in vitro* administration of the EZH2 inhibitor DZNep was capable of suppressing the proliferation potency of GSCs in an analysis of brain glioma [20, 63]. Furthermore, the suppression of EZH2 gene secretion was capable of reversing temozolomide (TMZ) resistance in patients with brain glioma [9, 63]. CSCs have been implicated in tumor recurrence after treatment, and their extreme chemoresistance and radiation resistance require alternative therapeutic schemes that are capable of effectively eradicating them (functional or physical) [20, 129].

Studies have demonstrated that c-MYC downregulation was capable of abolishing tumorigenicity exhibited by EZH2-depleted glioblastoma CSCs. It was further observed that complete loss of tumor-initiating capacity was capable of causing disruption in c-MYC in GBM CSCs (Figure 2) [20, 130, 131]. Furthermore, the knockdown of EZH2-mediated BMPR1B stimulated maintenance of CSCs in a subset of GBM, signifying that BMPR1B was responsible for the reduction in tumorigenic potential in EZH2-knockdown BT-CSC (Figure 2) [81]. Suvà et al. identified elevated secretion of EZH2 in tumor cells but no detectable secretion in the adjacent brain parenchyma in paraffin-embedded immunohistochemistry of GBM samples [20]. They indicated that pharmacologic as well as shRNA-mediated depletion of EZH2 in GBM CSCs decreases their capability to form new spheres *in vitro* and new tumors *in vivo* (Figure 2) [20].

TMZ is an oral chemotherapy agent which works by sensitizing the tumor cells to radiation with reduced side effects. TMZ has become the standard therapeutic option for GBM treatment [9, 132, 133]. Tumor recurrence and resistance remain key challenges with TMZ therapy although it has made an impact on the survival of several patients. Fan et al. established that silencing of EZH2 secretory levels was linked to a TMZ-resistant phenotype in GBM cells during gene secretory analysis of both the TMZ-sensitive and TMZ-resistant GBM cell lines [9]. Furthermore, EZH2 was extremely secreted in multidrug-resistant human glioblastoma cells U251/TMZ as well as U87/TMZ [9].

Fan et al. specified that administration of EZH2 siRNA into glioma cells effectively as well as quickly silenced EZH2 resulting in a reduction in mRNA and protein levels by about 70%, signifying the effective inhibitory effects of EZH2 siRNA [9]. They further indicated that EZH2 was capable of modulating cellular proliferation because downregulation of EZH2 was capable of decreasing the cell growth viability of U251/TMZ as well as U87/TMZ cells by about 30-40% [9]. Also, the knockdown of EZH2 resulted in reduction of MDR, MRP, and BCRP mRNA and protein levels leading to a decrease in efflux pump activity as well as augmented sensitivity to chemotherapy in GBM cells [9]. Thus, the anti-MDR influence of the EZH2 deletion was mediated by MDR, MRP, and BCRP [9].

Cheng and Xu demonstrated that the blockade of EZH2 secretion expressively repressed proliferation as well as tumorigenic efficiency of glioma cells [63]. They indicated that the blockade of EZH2 secretion was capable of downregulating the levels of numerous oncogenes including c-MYC and AKT [63]. Wu et al. further established that glioma patients with combined oversecretion of BMI1 as well as EZH2 proteins had the shortest overall survival. Moreover, secretion of BMI1 as well as EZH2 was observed as an independent prognostic factor for overall survival in glioma patients [120].

Natsume et al. demonstrated that the biological transformation between GSCs and differentiated non-GSCs is plastic in nature and escorted by gain or loss of PRC2-mediated H3K27me3 on pluripotency [110]. They further exhibited that EZH2 was extremely secreted in murine as well as human GSCs [110]. Also, administration of suberoylanilide hydroxamic acid triggered upregulation of PRC2 anticipated target genes, GSC disruption, and reduced secretion of EZH2 and stem cell marker CD133 [110]. Studies further demonstrated that the blockade of EZH2 secretion by shRNA was associated with a substantial reduction in the proliferation of glioma cells (Figure 2) [76, 119]. Furthermore, the blockade of EZH2 suppressed GBM tumor growth [76, 119].

Ahmad et al. established that treatment schemes targeting the disruption of EZH2-TERT-lipid metabolism interaction are capable of exhibiting intrinsic specificity for TERT mutant tumors as compared to TERT wild-type GBM tumors (Figure 2) [124]. Kim et al. demonstrated that the EZH2 and STAT3 signaling pathways are essential treatment targets for GBMs [64]. Kim et al. further established that targeting EZH2 may efficiently block oncogenic activities of both the EZH2 and STAT3 pathways (Figure 2) [64]. Yu et al. established that GSK343 was a feasible therapeutic approach as well as an imperative tool to understand the oncogenic function of EZH2 in glioma (Figure 2) [31]. Karlowee et al. discovered that notwithstanding varying age as well as tumor grades, EZH2 secretion was robust in high-grade glioma as well as in patients with a worse outcome [134]. Thus, EZH2 is a promising therapeutic as well as prognostic biomarker for the treatment of glioma.

## 9. Conclusions

EZH2 is well recognized as an essential modulator of cell invasion as well as metastasis in glioma. EZH2 oversecretion was implicated in the malfunction of several fundamental signaling pathways like Wnt/*β*-catenin signaling, Ras and NF-*κ*B signaling, PI3K/AKT signaling, *β*-adrenergic receptor signaling, and BMP as well as NOTCH signaling pathways. EZH2 was more secreted in GBM than in low-grade gliomas as well as extremely secreted in U251 and U87 human glioma cells. Thus, the blockade of EZH2 expression in glioma could be of therapeutic value for patients with glioma. The suppression of EZH2 gene secretion was capable of reversing TMZ resistance in patients with brain glioma. EZH2 is a promising therapeutic as well as prognostic biomarker for the treatment of glioma.

## Figures and Tables

**Table 1:** Immune/inflammatory factors and their influential effects on EZH2.

Immune/inflammatory factor	Effect of factors on EZH2 at a tumor milieu	Citations
Nutrients	Inhibitory	74
Hypoxia	Inhibitory	74
Acidic stress	Inhibitory	74
AXL	Inhibitory	53
n-Butylidenephthalide	Inhibitory	71
STAT3	Inhibitory	64, 79
INK4B-ARF-INK4A	Inhibitory	69, 80, 81
p57	Inhibitory	69, 80, 81
BMPR1B	Inhibitory	69, 80, 81
MyoD	Inhibitory	69, 80, 81
RUNX3	Inhibitory	69, 80, 81
CHD1	Inhibitory	82
TRAIL	Inhibitory	83, 84
FBO32	Inhibitory	83, 84
Vasohibin1	Inhibitory	85
MICU1	Inhibitory	86
Oxygen (O_2_)	Facilitatory	69
Deoxyglucose	Facilitatory	69
HIF1*α*	Facilitatory	69
PHD1-3	Facilitatory	69
EAF2	Inhibitory	69
siRNA	Inhibitory	87
DZNep	Inhibitory	87
GSK343	Inhibitory	31
CDKN2A	Inhibitory	12, 93
BRAF V600E	Facilitatory	93
iNOS	Inhibitory	94
TNF-*α*	Inhibitory	94
HCMV	Facilitatory	95
BMI1	Facilitatory	74

## Data Availability

No data was used in this paper.

## Abbreviations

DZNep: 3-Deazaneplanocin A
AEBP2: AE binding protein 2
As^3+^: Arsenic
BMI1: B cell-specific Moloney murine leukemia virus integration site 1
BMP: Bone morphogenetic protein
BMPR1B: Bone morphogenetic protein receptor 1B
CSCs: Cancer stem cells
CBX: Chromobox
FBXW1: *β*-TrCP
DNMTs: DNA methyltransferases
c-Cbl: Casitas B-lineage lymphoma
EZH2: Enhancer of zeste homolog 2
EED: Embryonic ectoderm development
E2Fs: E2 factors
ECM: Extracellular membrane
CHD1: E-cadherin gene
EMT: Epithelial-mesenchymal transition
EGBM: Epithelioid glioblastoma
EPCs: Endplate chondrocytes
ECAR: Extracellular acidification rate
FASN: Fatty acid synthase
GBM: Glioblastoma multiforme
GSCs: GBM stem-like cells
GPCR: G protein-coupled receptor
GLUT1: Glucose transporter 1
HMTase: Histone methyltransferase
HATs: Histone acetyltransferases
HDACs: Histone deacetylases
HIF1: Hypoxia-inducible factor 1
HCMV: Human cytomegalovirus
JARID2: Jumonji and AT-rich interaction domain containing 2
LI: Labeling index
MBD: Methyl-CpG-binding domain
MSCs: Mesenchymal stem cells
NSPC1: Nervous system Polycomb 1
TRAIL: TNF-related apoptosis-inducing ligand
BP: n-Butylidenephthalide
NICD1: NOTCH intracellular domain 1
NEAT1: Nuclear enriched abundant transcript 1
PH: Polyhomeotic
PcG: Polycomb group
PRC2: Polycomb repressive complex 2
Pcgf: Polycomb group ring finger
PCLs: Polycomblikes
PTMs: Posttranslational modifications
PCAF: P300/CBP-associated factor
pS21 EZH2: Phosphorylation of EZH2 at serine 21
pY461 EZH2: Phosphorylation at EZH2 Y641
PI3K: Phosphoinositide 3-kinase
PHD: Prolyl hydroxylases
RING: Ring finger protein
RbAp46/48: Retinoblastoma protein-associated protein 46/48
STAT: Signal transducer and activator of transcription
SMURF2: SMAD ubiquitination regulatory factor-2
SCF: Skp/cullin/F-box protein
SAH: S-Adenosylhomocysteine
SUZ12: Suppressor of zeste 12
Ser: Serine
Thr: Threonine
Tyr: Tyrosine
H3K27me3: Trimethylation of histone H3 on Lys 27
TET1: Ten-eleven translocation 1
TNF-*α*: Tumor necrosis factor-*α*
TERT: Telomerase reverse transcriptase
TMZ: Temozolomide
Wnt: Wingless-related integration site.
